# Controversial Role of Robot in Primary and Revisional Bariatric Surgery Procedures: Review of the Literature and Personal Experience

**DOI:** 10.3389/fsurg.2022.916652

**Published:** 2022-05-31

**Authors:** Giovanni Fantola, Enrico Moroni, Matteo Runfola, Emanuele Lai, Stefano Pintus, Pierpaolo Gallucci, Francesco Pennestrì, Marco Raffaelli

**Affiliations:** ^1^ Obesity Surgery Unit, Department of Surgery, ARNAS G. Brotzu, Cagliari, Italy; ^2^ Emergency Surgery Unit, Department of Surgery, ARNAS G. Brotzu, Cagliari, Italy; ^3^ Division of Endocrine and Metabolic Surgery, Fondazione Policlinico Universitario Agostino Gemelli IRCCS, Rome, Italy; ^4^ Division of Endocrine and Metabolic Surgery, Fondazione Policlinico Universitario Agostino Gemelli IRCCS, Università Cattolica del Sacro Cuore, Rome, Italy

**Keywords:** robotic surgery, daVinci, revisional surgery, gastric bypass, SADIS

## Abstract

Laparoscopy is the surgical standard of care for bariatric procedures; however, during the last two decades, the robotic approach has gained increasing interest. It is currently considered a safe and effective alternative to laparoscopy. This literature review investigates the role of the robotic approach for primary and revisional bariatric procedures, with the particular aim of comparing this technique with the standard-of-care laparoscopic approach. The feasibility of robotic dissection and suturing could have potential advantages: robotics may prevent the risk of leak and bleeding and other surgical complications, determining potential benefits in terms of operative time, length of hospital stay, and learning curve. Considering primary procedures, the literature reveals no advantages in robotic versus the laparoscopic approach for adjustable gastric banding and sleeve gastrectomy. Robotic Roux-en-Y gastric bypass is associated with a longer operative time and a shorter hospital length of stay than laparoscopy. The robotic approach in revisional surgery has been proven to be safe and effective. Despite the longer operative time, the robotic platform could achieve a lower bleeding rate compared with laparoscopy. The surgeon’s selection criteria related to referrals to the robotic approach of difficult-perceived cases could represent a bias. In conclusion, robotic surgery can be considered a safe and effective approach in both primary and revisional bariatric surgery, despite the lack of evidence to support its routine use in primary bariatric surgery. However, in revisional bariatric surgery and in surgical complex procedures, the robotic approach could have potential benefits in terms of surgical complications and learning curves.

## Introduction

Bariatric surgery has emerged as a safe and effective treatment (1) for morbid obesity, defined as BMI ≥ 40 or BMI ≥ 35 associated with obesity-related diseases. In 2019, 256,000 bariatric surgery operations have been performed in the USA (2), and in 2021, 22,469 bariatric surgery operations have been performed in Italy (3).

Minimally invasive bariatric surgical procedures have been associated with improved early postoperative outcomes in comparison with open approaches, with very low mortality and low morbidity rates (4).

Laparoscopic surgery is currently considered the standard of care in the bariatric surgery approach, as open surgery remains only as a rescue operation or in case of contraindications to laparoscopy. However, advanced laparoscopic skills are required to achieve adequate surgical outcomes after laparoscopic bariatric procedures (5). In some conditions, such as patients with prior abdominal surgery or in complex bariatric procedures, like biliopancreatic diversion with duodenal switch (BPDDS), the laparoscopic approach can be challenging. With this background, the use of the robotic platform in bariatric surgery has been growing over the last two decades (6).

The aim of this review is to assess the current literature about the advantages and disadvantages of primary and revisional robot-assisted bariatric procedures in comparison with the standard-of-care laparoscopic approach.

## Materials and Methods

A systematic literature search was performed on PubMed and Google Scholar. Searched terms included keywords [robotic] [bariatric] [surgery] [davinci] [rygb] [sleeve gastrectomy] [sadi-s] [revisional] [primary]. All titles and abstracts were assessed to select those focusing on robotic bariatric surgery. Subsequently, the full text of the selected trials was independently screened by the authors for eligibility. Inclusion criteria were comparative and non-comparative studies, including patients who underwent primary and revisional robotic bariatric surgery.

## Robotic Surgery

For two decades, the robotic platform has offered surgeons better instruments for performing minimally invasive procedures, allowing more difficult procedures to be performed, from esophageal to rectal resections, including bariatric surgery. Technical advantages include tridimensional viewing with a binocular camera, wristed graspers, dissectors, and staplers, providing both improved articulation and rotation, tremor filtering for more precise handling (7), allowing hand-sewn anastomosis (8), resulting in reduced fatigue (9), and improved surgical abilities (10) compared with laparoscopic surgery.

On the other hand, robotic surgery is still a subject of debate because of its longer learning curve, increased costs, and consequently reduced cost-effectiveness.

Indeed, cost is an important drawback in robotic surgery. Costs include robotic platform purchase, instruments with a limited life span, and annual service maintenance (20% of total cost/year). Overall, the DaVinci Robotic Surgical System with dual console and fourth arm costs about 4.5 million Euros on a 7-year pay-off period. Consequently, direct procedural costs are generally higher for robotic bariatric procedures in comparison with conventional laparoscopy. This overcost is on average about 2.3 times more expensive per patient (11). There are several factors that could reduce the cost per patient and improve cost-effectiveness. The number of robotic procedures per year using a specific robotic system may reduce the costs of initial purchase and annual maintenance. It is generally estimated that less than 250 procedures/year is not sufficient for a medical center to rationalize robotic overcosts. Decreasing the length of hospital and ICU stay could also be used to balance the overcosts in comparison with laparoscopic procedures. However, this remains to be proven using clear comparative data, because Bertoni et al. recently demonstrated that robotic surgery is only non-inferior to laparoscopic surgery (12).

## Primary Procedures

### Sleeve Gastrectomy

In 2000, Sudan et al. described the first case of robotic Sleeve Gastrectomy (SG) as the first part of robotic BPDDS (13). Current data have shown no clear postoperative improvements in comparison with the conventional laparoscopic approach. In this review, we only selected studies including more than 50 patients (Table 1). Romero et al. compared 134 patients who underwent a robotic SG with a systematic review of 3,148 patients after conventional laparoscopic sleeve (14). Sixty-three cases of anastomotic leaks (1.97%), 12 gastric strictures (0.43%), and 34 bleedings (1.21%) in the laparoscopic group were observed. No leak, no stricture, and only one bleeding (0.7%) were observed in the robotic group. The mean surgical time was longer in the robotic group (106 versus 94.5 min). However, the mean hospital stay was significantly shorter in the robotic group (2.2 versus 3.3 days). This study concluded that similar perioperative outcomes were observed in both approaches, although the robotic platform could provide a more comfortable environment for the operating surgeon.

**Table 1:** Literature review of robotic Sleeve Gastrectomy.

Study	*N*	Type	Operative time (min)	Major morbidity (%)	Conversion (%)	Los (day)
Romero (1)	134	CCT	106.6	3	0	2.2
Vilallonga (2)	100	CCT	108.18	5	0	4.3
Kannan (3)	103	CCT	110	0	0	4.2
Nasser (4) (*Revisional*)	1,077	RS	145.2	6.7%	0.3%	1.9

*CCT, clinical controlled trial; RS, retrospective study; NA, not available.*

Another comparative study analyzed 100 robotic versus 100 laparoscopic SG (15). It reported three leaks in the robotic group (3%) and four in the laparoscopic group (4%). The mean hospital stay was similar in both groups ranging from 3 to 4 days. This study concluded that the robotic approach was feasible, but cost issues and mean operative times may need to be improved. Kannan et al. (16) reported 108 patients (62 laparoscopic versus 46 robotic SG). This study showed no difference in terms of postoperative complication rates and mean operating time.

### Gastric By-Pass

In this literature review, we selected six clinical controlled trials, three case series, one systematic review, and one meta-analysis (Table 2). Only published series including more than 50 patients were considered. Ayloo et al. (17) reported a total of 135 RYGB (Roux-en-Y gastric bypass). Among them, 45 RYGB were performed laparoscopically and 90 were robot-assisted. This study showed no difference in terms of postoperative morbidity rates. Intraoperative data also showed a shorter operative time in robotic group patients (207 versus 227 min). Hagen et al. (18) reported 990 patients who underwent a gastric bypass from June 1997 to July 2010. There were 524 open, 323 laparoscopic, and 143 robotic cases. This study concluded that significantly fewer anastomotic complications were observed after open and robotic RYGB (0%) in comparison with laparoscopic RYGB (4.1%). However, this study is associated with potential bias due to its long inclusion period spanning over 13 years.

**Table 2:** Literature review of robotic Roux-en-Y Gastric Bypass.

Study	*N*	Type	Operative time (min)	Overall morbidity (%)	Major morbidity (%)	Conversion (%)	Los (day)
Sanchez (5)	50	RCT	130.8	0	0	1	2.72
Ayloo (6)	90	CCT	207	2.2	1.1	0	2
Fourman (7)	1,750	Systematic review	192	7.9	NA	NA	2.72–3
Hagen (8)	143	CCT	NA	16.1	NA	1.4	7.4
Tieu (9)	1,100	Case series	155	14	4.09	0	NA
Renaud (10)	154	Case series	141	33.1	11	2.6	NA
Ahmad (10)	172	CCT	155	NA	0	0	2.4
Smeenk (11)	100	CCT	117	5	3	0	2
Economopoulos (12)	5,155	Meta-analysis	NA	NA	NA	NA	NA
Myers (13)	100	CCT	144	NA	12	NA	2.1
Benizri (14)	100	CCT	130	24	13	0	9.3
Nasser (4) (Revisional)	1,230	RS	196.7	9.3%	NA	0.7	2.4

*RCT, randomized controlled trial; CCT, clinical controlled trial; RS, retrospective study; NA, not available.*

A review including a total of 18 studies was published by Fourman et al. (19). A total of 1,750 patients was included with a mean operative time of 192 min in robotic versus 173 min in laparoscopic group patients. The postoperative complication rate was similar in both groups (7.9% versus 8.6%). This review concluded that robotic group patients had fewer anastomotic leaks (4 versus 21 patients) and fewer anastomotic strictures (17 versus 40 patients).

In 2013, Benizri et al. (20) reported 200 RYGB performed by two experienced bariatric surgeons (100 robotic versus 100 conventional laparoscopic RYGB). This study showed a significantly higher postoperative morbidity rate in robotic group patients regarding surgical complications (13% versus 1%; *p* = 0.001). Consequently, more patients had a reoperation (9% versus 1%). However, intraoperative data showed a shorter mean operative time in the robotic group (130 versus 147 min). Overall, the mean hospital stay was longer in the robotic group (9.3 versus 6.7 days, *p* = 0.001). Later, Ahmad et al. (21) reported 173 laparoscopic versus 172 robotic RYGB procedures. No difference was observed in terms of intraoperative events, conversions to open procedures, leaks, strictures, returns to the operating room within 30 days, and mortality. Smeenk et al. (22) reported 100 laparoscopic versus 100 robotic RYGB procedures. The morbidity rate was 5% in both groups and major morbidity rates (Clavien-Dindo class 3–4) were similar (3% versus 1%; *p* = 0.62), and there was no mortality. The mean operative room time was 117 min in robotic versus 66 min in laparoscopic group patients (*p* < 0.05).

Non-comparative series was also used to evaluate robotic RYGB. Tieu et al. (23) included 1,100 patients with robotic RYGB from two high-volume medical centers. The ninety-day major complication rate was 4.09% (45 patients). Fantola et al. analyzed 302 consecutive robotic RYGB. In this study, the 60-day major complication rate (Clavien-Dindo score 3 or 4) and reoperation rate were 12.2% and 10.2%, respectively. Fourteen patients (4.6%) had a postoperative anastomotic leak requiring specific surgical management (24).

### SADI-S

Single Anastomosis Duodenal-ileostomy with Sleeve gastrectomy (SADI-S) is a novel surgical technique, emerging from the BDPDDS legacy, aiming to reduce BPDDS risks, while maintaining its efficacy as one of the most effective interventions. It is considered a valid alternative to BPDDS, associated with a lower complication rate (25). It has also usually been considered an alternative to one-anastomosis gastric bypass (OAGB), more challenging from a technical point of view, but associated with a lower risk of complications.

Very few studies reported data from robotic SADI-S procedures. Nelson et al. reported a case series of robotic procedures (also including 10 laparoscopic procedures) and demonstrated that the robotic approach is safe and effective with an acceptable risk–benefit ratio but does not offer a comparison with the laparoscopic or open approach.

## Revisional Surgery

In the last few years, the number of reoperative procedures is rapidly increasing, as every year, more and more primary bariatric procedures are performed.

Reoperative bariatric surgery has been classified by Brethauer et al. (26) into the following:

• Conversion: procedures that change from an index procedure to a different type of procedure.
• Corrective: procedures addressing complications or incomplete treatment effect of a previous bariatric operation.
• Reversal: procedures that restore original anatomy.

Insufficient weight loss or weight regain, initial defect or late-surgical complication (e.g., GERD (27)), excessive weight loss, and malnutrition are an indication for revisional bariatric surgery (28) that must be taken into account in a multidisciplinary setting that includes surgical, nutritional, and psychological risks and benefits (29).

Revisional surgery has been associated with poorer outcomes in both RYGB (30), with increased intraoperative blood loss, longer operative time, longer adhesiolysis, longer hospital length of stay, higher rate of splenic injury, anastomotic leakage, and surgical site infections in comparison with primary RYGB, and in revisional SG compared with primary SG (31), with longer operative time, higher complication rate, and anastomotic leakage rate.

Due to increased operative difficulty, because revisional surgery is more challenging and requires a higher level of technical skills, the robotic platform may be considered for better visualization, dissection, and a wider range of movement provided by robotic instruments that could provide a better outcome to bariatric revisional procedures (32).

Clapp et al. (33) systematically analyzed revisional weight loss robotic surgery from the MBSAQIP database spanning from 2015 to 2016 and compared them with laparoscopic revisional procedures. One thousand nine hundred twenty-nine robotic procedures and 35,998 laparoscopic revisional procedures were found. Statistical analysis showed a longer operative time, prolonged hospital length of stay, and higher ICU admission rate in the robotic group than in the laparoscopic group. Other parameters taken into account showed no differences, which led the authors to state that no difference exists in postoperative complications. As a higher number of anastomosis revisions were done in the robotic group, the authors hypothesized that a selection bias could exist, with surgeons preferring robotic surgery in high-difficulty-perceived procedures.

A comparative analysis from Nasser et al. (34) (Tables 1, 2) taking into account procedures from the MBSAQIP database performed between 2015 and 2017 analyzed a series of revisional procedures, comparing the laparoscopic and robotic approaches.

As for revisional SG, 15,935 procedures were laparoscopic and 1,077 were robotic. Demographic characteristics were homogeneous in the two groups, and the authors found differences in terms of longer operative time in the robotic group, higher incidence of intraoperative adhesiolysis in the robotic group, higher incidence of overall morbidity in the robotic group, and higher incidence of ICU admission, sepsis, and organ-space SSI in the robotic group. No differences were found in terms of other parameters.

As for revisional RYGB, 11,212 procedures were laparoscopic and 1,230 were robotic. Demographic characteristics were homogeneous, a higher rate of drainage placement was found in the laparoscopic group, the operative time was longer in the robotic group, respiratory complications, postoperative pneumonia, and superficial SSI were higher in the laparoscopic group, and the incidence of bleeding requiring transfusion was higher in the laparoscopic group. No differences were found in terms of other parameters. Study limitation embraced a possible surgeon’s selection bias, as challenging cases might have been purposely selected for the robotic or laparoscopic group, and the MBSAQIP database provides postoperative data only up to 30 days, from a variety of heterogeneous centers.

Vilallonga et al. (35) presented a 3-case report of robotic revisional SADI-S (36) secondary to SG. In this study (35), the authors conclude the feasibility of robotic revisional SADI-S and its advantages over RYGB, as the Billroth-2 style duodenal-ileal-anastomosis might reduce operative time and postoperative complications.

A single-center study from King et al. (37) including 167 revisional procedures (115 laparoscopic, 52 robotic) demonstrated a reduced major complication rate in the robotic group than in the laparoscopic group (1 [1.9%] versus 6 [5.2%], *p* > 0.05) and a shorter hospital length of stay in the robotic group than in the other group (40.2 versus 62.6 h, respectively, *p* < 0.05), with no differences in minor complications, blood loss, and readmission rates.

Rebecchi et al. (38), reporting their results from a single-center study, performed a cost-related analysis of revisional robotic RYGB performed with the DaVinci Robotic Surgical System. The mean cost was found to be 14,334.70€ ± 2,920.40€, but they demonstrated that specific-robot expenses decreased in the last 10 cases of the study (9,708.50€ ± 515.70€) compared with the first 30 cases (12,168.70€ ± 1,438.40€), as the total cost for a laparoscopy procedure was 3,176.50€ ± 850.50€.

The increased costs of the robotic procedure have to be addressed in specific semidisposable instruments and robotic maintenance fees, even though the clinical benefits could overcome increased costs.

## Authors’ Personal Experience

Between the period January 2013 and March 2022, over 3,659 bariatric procedures (403—11.6% revisional ones) were performed at the Division of Endocrine and Metabolic Surgery (Fondazione Policlinico Universitario Agostino Gemelli IRCCS, Rome, Italy). Sixty-seven (1.8%) patients were scheduled for a robotic approach. Specifically, 37 RYGB, 23 SADI-S, 6 OAGB, and one BPD were performed.

The first robotic procedure, an RYGB, was performed in January 2013, while the first robotic SADI-S was performed in July 2016. However, the robotic approach was implemented starting from 2019, with a relative reduction in the number of performed procedures during the first phases of the COVID-19 pandemic (2020–2021).

As previously reported, 37 robotic-assisted RYGBs were performed. The median operative time was 201 min. We experienced only one postoperative surgical complication, a gastroenteric anastomotic leak, in a super-super-obese patient (BMI ≥ 60 kg/m^2^), which required surgical exploration (Clavien-Dindo IIIb). The subsequent postoperative course was uneventful, and the patient was discharged after 14 days.

In addition, 23 SADI-S were performed, in 22 cases as a primary procedure and in 1 case as a revisional operation. Recently, we reported our personal experience (currently under peer review) comparing the robotic and laparoscopic approaches in SADI-S, using propensity score matching analysis to avoid potential bias selection. The median operative time for robotic SADI-S was 191.5 min with the docking step requiring approximately 10 min. Using the CUSUM method to analyze the learning curves of the different approaches (laparoscopic versus robotic) for SADI-S, we observed a significant reduction in operating time after the first seven cases for the robotic SADI-S and after the first 47 cases for the laparoscopic approach. Thus, the use of the robotic platform reduces the time required to complete the learning curve for these challenging procedures. In addition, our analysis highlights that the laparoscopic and robotic approaches for SADI-S are comparable in terms of safety for reoperative and postoperative complications.

Furthermore, we performed six OAGBs (five primary procedures and one revisional) with a median operative time of 191 min. We chose this procedure at the beginning of our experience with the robotic platform in obese patients before the introduction of SADI-S in our clinical practice. For the same reason, we also performed a DPD with an operative time of 240 min.

From July 2021, the Obesity Surgery Unit (ARNAS G. Brotzu, Cagliari, Italy) started a robotic bariatric program. Eight robotic-assisted RYGB were performed. The median operative time was 240 min, with an improvement after the first five cases. One patient was reoperated because of alimentary limb occlusion due to jejuno-jejunal kinking.

Overall, after combining both series, no intraoperative deaths occurred in our robotic experience. No conversion was necessary, either to open or laparoscopic surgery. No 30-day mortality was recorded.

In our experience, the main indications for the robotic approach are challenging for bariatric patients. “Challenging cases” are a clinical characteristic that cannot be assessed by a single parameter but is determined by the presence of one or more of these conditions: patients with a BMI ≥ 50 kg/m^2^, especially if they are male and/or with a previous major abdominal surgery. Furthermore, according to other authors, we trust that the robotic platform can add value to these patients by reducing the number of two-stage procedures.

## Discussion

Laparoscopy has been the standard of care in choosing a surgical approach in bariatric surgery since the last two decades and has contributed to the large diffusion of bariatric procedures worldwide. In this context, robotic surgery remains controversial.

In our review, we analyzed some of the most performed procedures, considering both primary and revisional procedures, and we observed that in primary surgery, robotic surgery is safe and effective but offers no advantages in perioperative care compared with laparoscopic surgery. However, in revisional surgery, which has a higher rate of postoperative complications due to adhesions and altered anatomy and, therefore, requiring higher laparoscopic skills, the robotic approach could have a primary role. This was proved by Nasser et al. (34), who demonstrated a reduced complication rate in revisional robotic RYGB compared with the laparoscopic approach.

It must also be considered that in most studies taken into account, there was no patient randomization, and patients who were perceived more challenging by the surgeon were assigned to robotic surgery, with a clear selection bias. These obviously included superobese patients (BMI > 50 kg/m^2^) in whom fine dissection and, especially, suturing could be particularly challenging without the robotic platform. Further studies comparing a similar group of patients, eventually by randomization, are, thus, necessary in order to draw a definitive conclusion in this regard.

On the other hand, as most of the bariatric procedures are performed on a single abdominal quadrant, robotic platform benefits could be less emphasized compared with other surgical fields and have a longer learning curve and longer operative time than laparoscopy (39). However, the authors’ personal experience has demonstrated that the robotic platform is able to reduce the learning curve for a single, complex procedure, as SADI-S does.

Cost-effectiveness has been, since its adoption, one of the main drawbacks in robotic surgery, as DRG reimburses might be insufficient to cover robotic extra costs (40). A cost analysis by Khorgami et al. (41) demonstrated that robotic surgery is the factor associated with the highest impact on extra costs in bariatric surgery.

However, as Hagen et al. (42) demonstrated that laparoscopic staplers might reduce robotic costs compared with robotic staplers, robotic costs can be further mitigated with avoidance of staplers with handsewn anastomosis, which showed a reduced complication rate compared with laparoscopic-stapled anastomosis (18) and, therefore, a further cost mitigation. Moreover, as a robotic platform enhances the surgeon’s ability in sewing compared with laparoscopy, further studies are needed to compare handsewn anastomosis and stapled mechanical anastomosis, considering that handsewn anastomosis is tailored, is opposed to standardized staplers, and the anastomotic stricture rate tends to be lower with a handsewn technique (43), and that could be most useful in complex procedures (i.e., SADI-S).

Most studies found in the current literature were based on the DaVinci Robotic Surgical System, while no data were found in regard to other robotic platforms.

More studies are, however, necessary to address the efficacy and sustainability of the robotic approach, as most studies to date have lacked randomization and, therefore, have a selection bias that has prevented a proper data evaluation. It should also be noted that a fully trained surgeon in robotic bariatric surgery, and, therefore, able to deal with challenging cases intended for robotic surgery, has to complete a learning curve that, especially in the initial stages, cannot be based on challenging cases and, therefore, includes primary bariatric procedures that currently, due to increased costs, are not routinely performed with the robotic platform but with laparoscopy instead.

## Conclusion

Robotic surgery remains a valid surgical approach in treating morbid obesity, but, because of its inflated costs, it cannot be a first-choice option as its advantages over laparoscopy are restricted to surgeon ergonomics and not to patient perioperative care and, therefore, it is not financially sustainable. However, in selected cases, including revisional cases and superobese patients, robotic surgery could have a potential role in reducing postoperative complications and improving perioperative care, and, therefore, it also could be economically sustainable.

## Author Contributions

GF contributed to primary procedures, EL, MR, SP, EM, FP, and PG contributed to introduction, revisional procedures, and discussion, and MR reviewed the manuscript.
